# Molecular Compressive Force Sensor for Mapping Forces at the Cell–Substrate Interface

**DOI:** 10.1021/jacs.3c13648

**Published:** 2024-02-28

**Authors:** Sarah Al Abdullatif, Steven Narum, Yuesong Hu, Jhordan Rogers, Rachel Fitzgerald, Khalid Salaita

**Affiliations:** † Department of Chemistry, Emory University, 1515 Dickey Drive, Atlanta, Georgia 30322, United States; ‡ Department of Biomedical Engineering, Georgia Institute of Technology and Emory University, Atlanta, Georgia 30322, United States

## Abstract

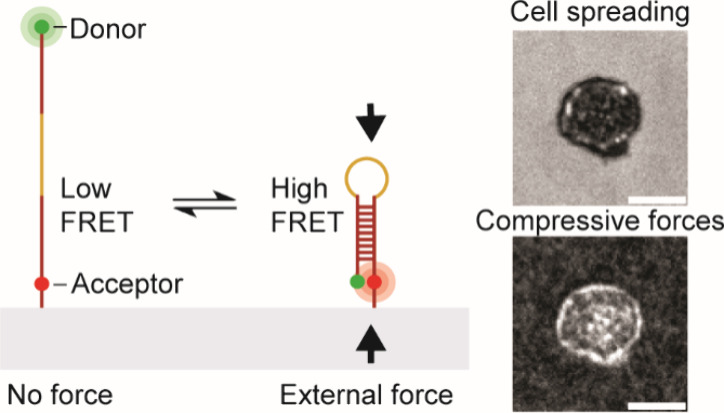

Mechanical forces are crucial for biological processes such as T cell antigen recognition. A suite of molecular tension probes to measure pulling forces have been reported over the past decade; however, there are no reports of molecular probes for measuring compressive forces, representing a gap in the current mechanobiology toolbox. To address this gap, we report a molecular compression reporter using pseudostable hairpins (M-CRUSH). The design principle was based on a pseudostable DNA structure that folds in response to an external compressive force. We created a library of DNA stem-loop hairpins with varying thermodynamic stability, and then used Förster Resonance Energy Transfer (FRET) to quantify hairpin folding stability as a function of temperature and crowding. We identified an optimal pseudostable DNA hairpin highly sensitive to molecular crowding that displayed a shift in melting temperature (*T*_m_) of 7 °C in response to a PEG crowding agent. When immobilized on surfaces, this optimized DNA hairpin showed a 29 ± 6% increase in FRET index in response to 25% w/w PEG 8K. As a proof-of-concept demonstration, we employed M-CRUSH to map the compressive forces generated by primary naïve T cells. We noted dynamic compressive forces that were highly sensitive to antigen presentation and coreceptor engagement. Importantly, mechanical forces are generated by cytoskeletal protrusions caused by acto-myosin activity. This was confirmed by treating cells with cytoskeletal inhibitors, which resulted in a lower FRET response when compared to untreated cells. Furthermore, we showed that M-CRUSH signal is dependent on probe density with greater density probes showing enhanced signal. Finally, we demonstrated that M-CRUSH probes are modular and can be applied to different cell types by displaying a compressive signal observed under human platelets. M-CRUSH offers a powerful tool to complement tension sensors and map out compressive forces in living systems.

## Introduction

Mechanical forces are a key component to many biological processes, including cancer invasion, stem cell differentiation, and T cell antigen recognition.^1−3^ Cells can sense mechanical forces at the molecular scale through various mechanisms that include exposing cryptic sites within proteins or alternatively by activating enzymatic processes. The forces experienced by mechanosensitive proteins may be intrinsically generated by the cell’s own cytoskeleton or externally generated such as the case for shear flows. Accordingly, mechanical cues are transduced into biochemical signals which aid the cell in making decisions involving activation, migration, proliferation, differentiation, and apoptosis.^3−6^ For example, during the antigen search process, T cells use microvilli which protrude from their surface to push and pull on neighboring cells.^7^ This mechanical activity is thought to play a role in tuning triggering of the T cell receptor (TCR) upon antigen engagement, and hence boosting the sensitivity in identifying and destroying cancer cells or infected cells that present foreign antigen.^3,8^ Given the important role that molecular forces play in biochemical pathways, there is growing interest in developing tools for quantifying and mapping biophysical forces in living systems.

Previous work has resulted in the development of genetically encoded tension sensors as well as molecular tension probes which report receptor–ligand pulling forces at pN resolution.^5,9−11^ The majority of these sensors are composed of a fluorophore and quencher separated by a flexible linker, such as a protein or DNA. When the linker is in its resting state, donor fluorescence is quenched. Upon the application of a tensile force, the linker is stretched, separating the fluorophore and quencher and resulting in a “turn-on” fluorescence signal for the donor or a FRET “turn-off” response.^9−12^ The sensitivity and force threshold of the probe can be tuned through a range of parameters including the choice of linker, geometry, folding stability, etc.^3,5,6^ Through studying the mechanical interactions of cells, researchers have revealed transduction mechanisms for integrin receptor activation, B-cell receptor antigen recognition, and Notch-Delta signaling among other mechanosensitive pathways.^3,6,9^

Despite the wide array of tension sensors reported, to the best of our knowledge, there are currently no probes available for measuring molecular *compressive* pushing forces exerted by cells. This is surprising as compressive forces may play equally important biochemical roles as tension forces.^13,14^ Previous attempts to study compressive forces exerted by cells included placing cells onto a substrate and measuring displacement of the substrate layer, through either elastic resonance interference stress microscopy (ERISM) or tracking fluorescent beads in the substrate.^15,16^ However, these techniques are not able to sense molecular scale forces, since groups of molecules are required to exert forces in coordination to deform a large area of the substrate, which also limits their spatial resolution.

Meanwhile, molecular scale *crowding* forces have been studied in biological systems using Förster Resonance Energy Transfer (FRET) to measure the distance between fluorophores as a function of excluded volume due to high crowder concentrations.^17^ In recent work, DNA folding dynamics have also been studied in the presence of molecular crowders. It is shown that molecular crowding stabilizes the folded conformation of a DNA hairpin.^18−20^ For example, pN scale depletion forces generated by molecular crowding have been quantified using a programmable DNA origami probe.^21^

**Scheme 1:**
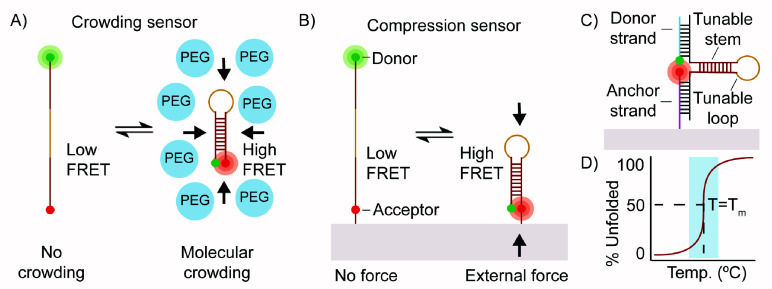
(A and B) Transition of DNA Hairpin from the Unfolded State (No Force, Low FRET) to the Folded State (Compressive Force, High FRET) in Response to Crowding Agents and External Compressive Force; (C) M-CRUSH Probes Are Comprised of Three DNA Strands: Donor Strand, Anchor Strand, and Tunable Stem-Loop; (D) Idealized Hairpin Melting Transition The shaded region indicates the ideal temperature range for generating maximum change in FRET in response to compressive force and crowding.

This inspired us to apply the principles of molecular crowding, which is the isotropic concentration induced reduction in the entropic freedom of a biomolecule or polymer, in the design of an interfacial molecular compression sensor (Scheme 1A and B). We define compression as a directional reduction of entropic freedom mediated by active mechanical forces at interfaces. In this way, compressive forces can fall under the umbrella of crowding.

The objective of this work is to develop tools to measure molecular compressive forces generated by living cells. Specifically, we created a molecular compression reporter using pseudo-stable hairpins (M-CRUSH). Since there is literature precedent showing crowding induced folding of DNA hairpins, we decided to leverage hairpin folding dynamics in the design of our interfacial probes. We employed FRET-based readout given its noninvasive, facile, and widespread use in cell and molecular biology. The probe consists of three DNA strands and a FRET pair. The responsive DNA strand contains a tunable stem-loop hairpin domain that allows for control of the Δ*G* of folding. The responsive strand is hybridized to a donor strand and an anchor strand modified with an acceptor dye. These three oligonucleotides assemble into a functional compression sensor that is immobilized onto a surface (Scheme 1C).

## Results and Discussion

In order to generate the largest change in FRET, we aimed to design the M-CRUSH probe such that it is mostly unfolded at room temperature in buffer that mimics the ionic strength of physiological conditions (Scheme 1D). We hypothesized that a hairpin that is pseudostable at room temperature would only require a small amount compressive force to induce folding, resulting in an enhanced increase in FRET signal. Conversely, if the hairpin melting temperature (*T*_m_) is ≫RT or ≪RT (i.e., highly stable or highly unstable at RT), then the probe will be insensitive to external forces and changes in FRET would be minimal. To test this hypothesis, a library of DNA hairpins were synthesized and evaluated (Table 1). The stability of each hairpin was tuned using a range of parameters including the size of the stem and loop region, GC content, and mismatches in base pairings. NUPACK^22^ was used to simulate the change in free energy (Δ*G*_*folding*_) and fraction of base pairs folded at room temperature for each of the hairpins synthesized. The *T*_m_ and Δ*G*_*folding*_ of each hairpin were determined experimentally in 1× PBS at 100 nM DNA concentration. Throughout this work, each hairpin was identified by its experimental melting temperature (*T*_m_).

**Table 1:**
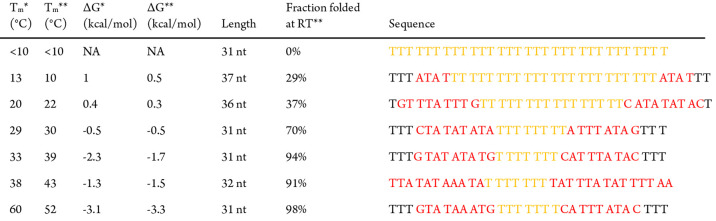
Library of DNA Sequences^a^

a The sequences display the stem-loop region ignoring the arms, and listed according to stability. The stem region is shown in red and the loop is in yellow. *T*_m_* and ΔG* were measured in 1× PBS at 100 nM concentration, while *T*_m_**, ΔG**, and fraction folded** were calculated using NUPACK in 1× PBS and at 100 nM DNA at room temperature.

**Figure 1:**
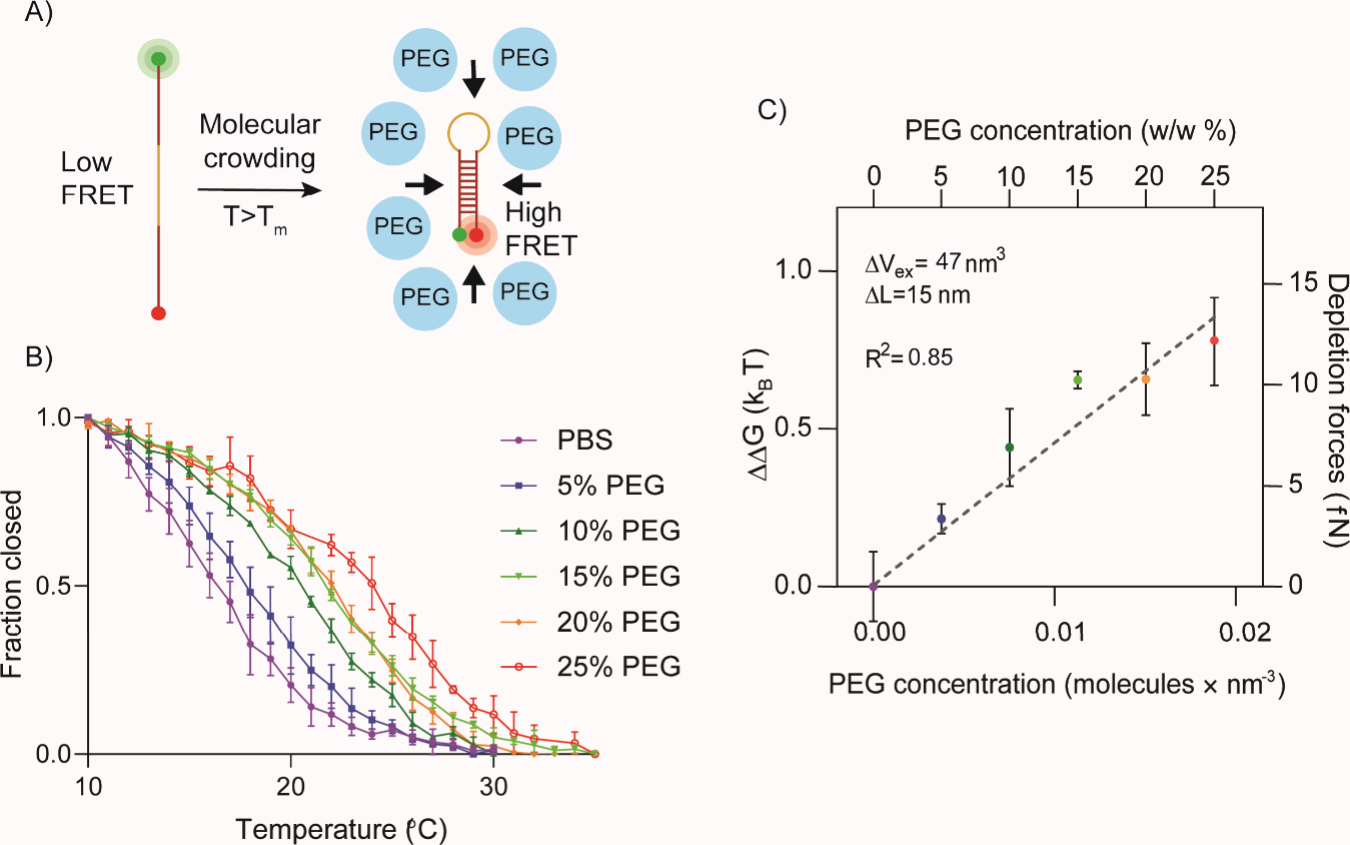
(A) Transition of DNA hairpin from unfolded (no crowding, high temperature, low FRET) to folded (molecular crowding or low temperature, high FRET). (B) Melting transitions for 20 °C hairpin in increasingly crowded conditions. Higher PEG concentration stabilizes the folded state, resulting in a higher *T*_m_. (C) Change in free energy (ΔΔ*G*) between the unfolded and folded state as a function of PEG concentration (1 kT = 0.59 kcal/mol). The depletion force (right axis) is determined from ΔΔ*G*/Δ*L* and reveals femtonewton scale force measurement resolution. Error bars represent the standard error of the mean from triplicate thermal melt measurements.

After obtaining the melting curve for each hairpin, their thermodynamic properties were inferred using a van’t Hoff analysis (Figure S3). We focused our investigation on the 20 °C hairpin because we predicted that it would be the most sensitive molecular compression probe (Figure 1A and B). We measured its melting transition as a function of crowding using 8000 g/mol PEG and found that crowding could drastically enhance stability, with its *T*_m_ increasing by up to 7 °C in 25% PEG (Figure 1B). To estimate the forces driving M-CRUSH folding, we inferred the change in free energy (ΔΔ*G*_*folding*_^*PEG*^) as a function of PEG concentration (Figure 1C). The slope of the resulting plot gives the change in excluded volume (Δ*V*_ex_) as indicated in eq 1.
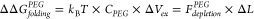
1

We then divided Δ*V*_ex_ by the cross-sectional area of a DNA duplex (assuming 2.0 nm diameter) to determine Δ*L* which matches the expected mean end-to-end length of the single stranded region. The depletion force can also be quantified using eq 1 and is estimated at the tens of femtonewtons scale. This estimate is lower than PEG-mediated crowding forces reported by Castro et al., but in their case larger DNA origami structures with greater Δ*V*_ex_ were used which may explain the different crowding forces.^21^

**Figure 2:**
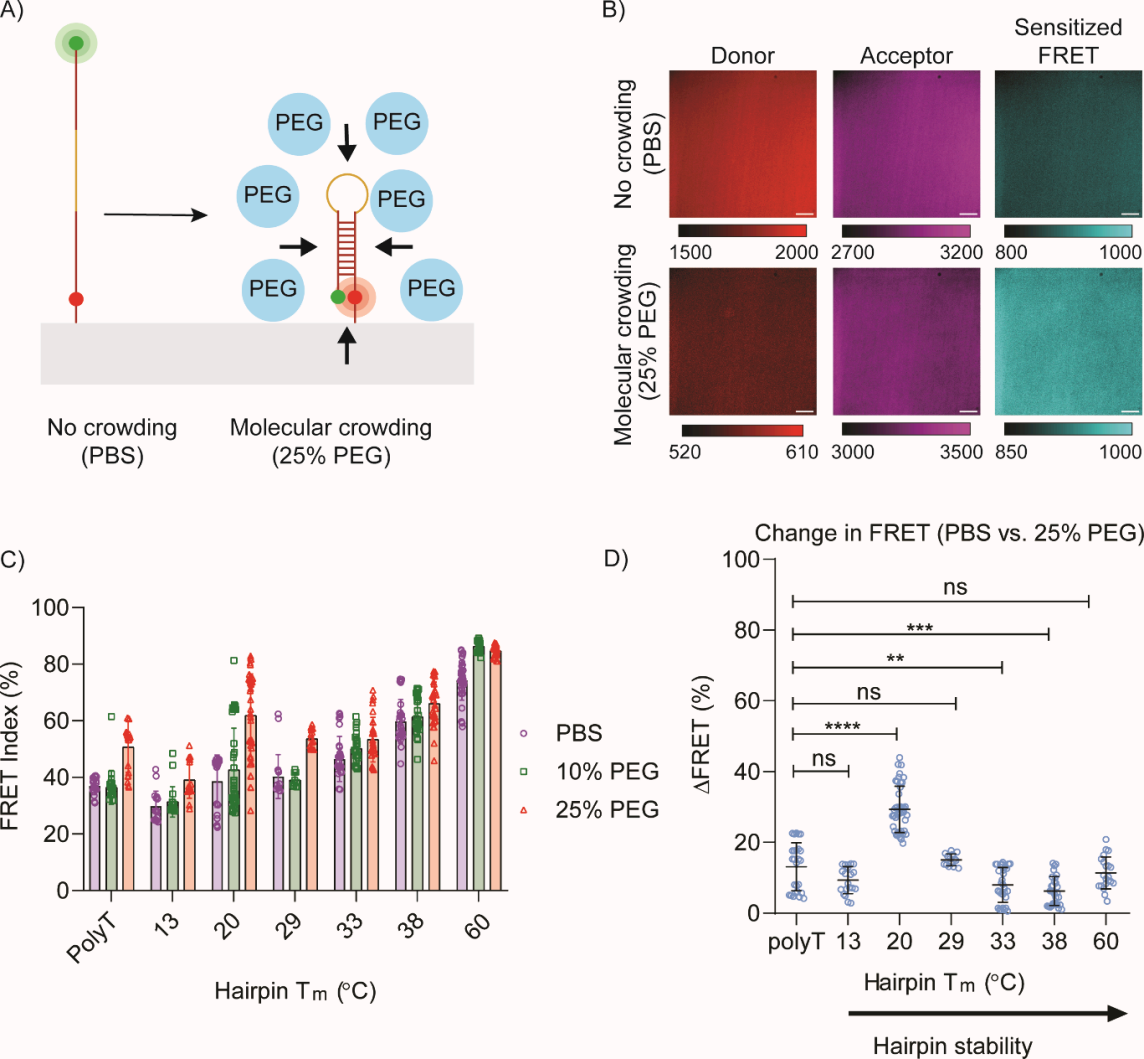
(A) Schematic representing PEG-induced folding of immobilized sensors. (B) Representative images for immobilized 20 °C hairpin in donor, acceptor, and sensitized FRET channels. Scale bar = 10 μm. The top row shows the hairpin in the absence of molecular crowding (1× PBS) while the bottom row shows the hairpin in a crowded environment (25% PEG). (C) FRET index measurements performed on library of immobilized DNA hairpins in PBS buffer (purple circle), 10% (w/w) PEG (green square), and 25% (w/w) PEG (red triangle). As PEG concentration increases, the FRET index increases; however, this effect is greatest in pseudostable hairpins. (D) Plot of ΔFRET in response to PEG crowding as analyzed from data in (C). Note that the 20 °C hairpin showed the highest ΔFRET. Error bars represent the standard error of the mean from triplicate surface measurements. *P* values are indicated as follows: 0.01(**), 0.001 (***), <0.0001 (****).

To investigate probe response on surfaces, we next immobilized the sensor onto glass surfaces and imaged in 3 channels: donor, acceptor, and sensitized FRET (Figure 2A). Folding was quantified by using the FRET index, which is calculated using eq 2 and uses the bleedthrough subtracted donor intensity and sensitized FRET intensity (Figure 2B).
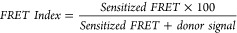
2

For each DNA sequence, three conditions were tested: no molecular crowding (PBS), low molecular crowding (10% PEG), and high molecular crowding (25% PEG). As expected, all DNA hairpins showed an increase in FRET upon the addition of the crowding agent (Figure 2C). However, the greatest crowding-induced change in FRET index of 29 ± 6% was observed for the 20 °C hairpin (Figure 2D). Similar trends were also observed when this experiment was repeated in solution, without immobilizing the hairpins on glass surfaces (Figure S4). These results support our hypothesis that a pseudostable hairpin would be highly sensitive to crowding forces. Changes in FRET for the 20 °C hairpin were further validated using Fluorescence Lifetime Imaging Microscopy (FLIM) as shown in Figure S5. Given these results, the 20 °C hairpin was selected as the primary sequence for the M-CRUSH probe.

**Figure 3:**
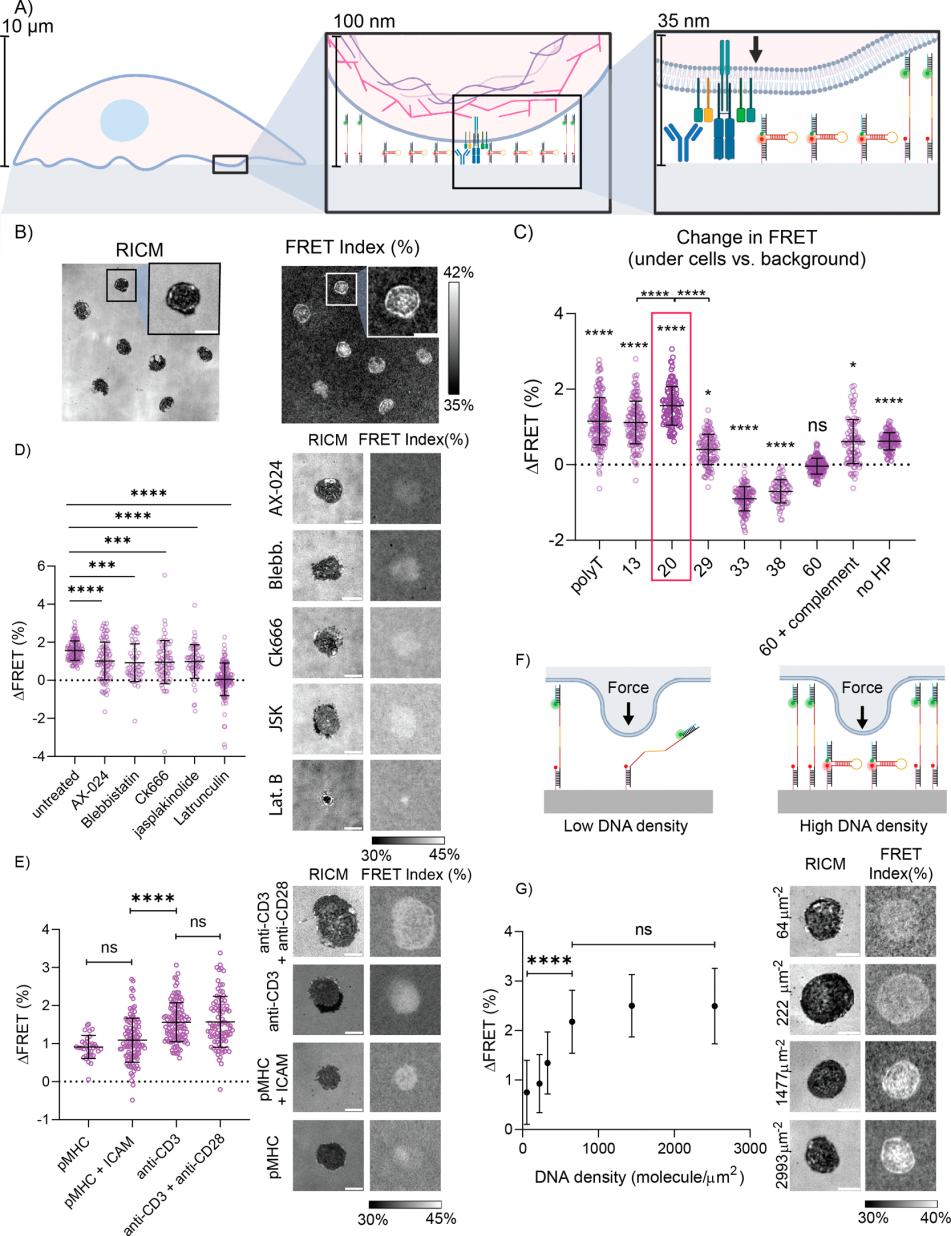
(A) Schematic showing experimental setup. T cells are plated on modified glass slides decorated with M-CRUSH probes as well as anti-CD3 antibody. (B) Representative images showing RICM (left) and FRET index (right). (C) Change in FRET for library of tested hairpins. Labels above each set of data points represent the significance of ΔFRET from the background FRET. Labels above the brackets indicate the statistical difference between groups. The 20 °C hairpin shows the highest increase in FRET under cells (highlighted in red box). (D) Screening of T cells pretreated with acto-myosin inhibiting drugs for 10–15 min: AX-024 (500 nM), blebbistatin (50 μM), ck666 (50 μM), jasplakinolide (500 nM), and latrunculin B (5 μM). A decrease in FRET is observed in treated cells. (E) Change in FRET index for 20 °C hairpin when pMHC, pMHC+ICAM, anti-CD3, and anti-CD3+anti- CD28 ligands are presented on the surface. Anti-CD3 ligands resulted in the largest change in FRET index. (F) Schematic showing proposed role of probe density on response. (G) Plot displays relationship between M-CRUSH probe density and change in FRET index under T cells. Representative RICM and FRET index images for T cells seeded on anti-CD3 surface presenting M-CRUSH probes at different densities. Scale bar = 5 μm in all representative images. Each data point represents a single cell. Error bars show the standard error of the mean from *n* = 3 biological replicates. *P* values are indicated as follows: 0.05 (*), 0.001 (***), <0.0001 (****).

Next, we decided to test the M-CRUSH probe with transgenic T cells that express a monoclonal population of TCRs which respond to a cognate antigen derived from ovalbumin (OVA). Naïve CD8+ T cells were isolated and plated onto glass slides presenting both M-CRUSH and the TCR binding antibody anti-CD3 (Figure 3A). Note that cell–surface interactions were ligand–receptor specific, and surfaces lacking antibodies failed to allow any detectable cell adhesion, spreading, or activation (Figure S6). After allowing the cells to spread on the surface for 25 min they were imaged in the same fluorescence channels described previously and the FRET index was quantified using eq 2. Cell spreading was also visualized using reflection interference contrast microscopy (RICM). We observed an increase in FRET index for the regions underneath cells compared to the surrounding regions for the vast majority of T cells that spread as noted from the RICM channel. Moreover, line scan analysis showed an up to 6% increase in FRET index at the perimeter of the cell-surface contact zone as well as in a subset of puncta under the cell (Figure S7). Timelapse imaging of the initial T cell–substrate contact showed dynamically growing FRET signal that tracked with cell spreading (Figure S8, red arrow). Timelapse imaging also revealed weakening FRET signal in other cells that displayed reduced surface contact (Figure S8, blue arrow). These data indicate that the compressive forces generated by T cells are highly dynamic.

We also tested a library of M-CRUSH probes created using different DNA hairpins (Table 1) and measured the FRET index across this library in triplicate cell measurements (Figure 3C). As controls, we also tested the 60 °C probe hybridized to a complementary DNA strand (60+complement) and prepared a surface presenting a binary mixture of ssDNA-Cy3B and ssDNA-Atto647N oligos at equal concentrations (no HP). Consistent with the PEG crowding results, we found that the 20 °C hairpin showed the greatest change in FRET, with the average FRET under the entire cell–substrate contact zone at 1.6 ± 0.5% greater than the background (Figure 3C). The 20 °C hairpin M-CRUSH probe performed better than all the highly stable hairpins as these showed a dampened response or in some cases a loss in FRET index which may be due to local changes in index of refraction.^23,24^ The controls also showed a dampened FRET response, while probes created using highly unstable DNA structures (13 °C and polyT) showed some sensitivity to compressive force likely due to adopting random coil conformations.

Compressive forces generated by the cell are likely driven by protrusions of the cytoskeleton caused by actin polymerization. To test this hypothesis, we measured the M-CRUSH response in cells pretreated with well-documented cytoskeletal inhibitors. In particular, we used AX-024, an inhibitor of TCR-Nck-WAVE complex interactions;^25^ blebbistatin (blebb.), an inhibitor of myosin II;^26^ Ck666, which binds to Arp2/3 and prevents the nucleation of branched actin filaments;^27^ jasplakinolide (JSK), which destabilizes actin filaments in vivo;^28^ and latrunculin B, an inhibitor of F-actin polymerization.^29^ We found that each of these drugs results in the depletion of the compressive force (Figure 3D). The activity of each of these agents was confirmed by observing a decrease in the cell spreading area (Figure S9). These results further validate the role of cytoskeletal dynamics in mediating M-CRUSH signal. The contributions of the cytoskeleton were further confirmed in experiments where cells were fixed in solution prior to plating on surfaces (Figure S10). Fixed cells bound to antibodies on the surface; however, no cell spreading nor change in FRET was noted underneath cells. These results indicate that antibody binding alone does not result in an increase in FRET.

To further investigate how the M-CRUSH probe responds to the activation state of T cells, we challenged cells using additional ligands that were reported to enhance T cell mechanotransduction and investigated their roles in regulating compression forces. Four ligands were tested on surfaces with the M-CRUSH probe. The first was the peptide major histocompatibility complex (pMHC) which was loaded with the cognate SIINFEKL peptide. The second was pMHC with the ICAM adhesion ligand. We also tested two anti-CD3 surfaces, one that was a reference surface and another that included T cells with CD28 coreceptor stimulation using anti-CD28 antibodies. The FRET index was measured under cells in these four groups, and we found that the greatest compression forces were generated in the presence of anti-CD3 regardless of CD28 stimulation. This is likely a reflection of the high binding affinity between the TCR and anti-CD3^30^ which is likely the major contributor to generating compressive forces (Figure 3E).

Importantly, the presentation of pMHC along with ICAM triggered a migratory T cell phenotype, as has been reported in the past.^31,32^ As cells migrate across the surface, the FRET signal also moved along with the cell contact zone (Figure S11). This further validates the conclusion that the observed compression signal is cell associated and transient.

Interestingly, the observed ΔFRET signal driven using molecular crowding was significantly greater than that mediated by cellular forces. This may be due to the isotropic nature of crowding in contrast to compressive forces that are primarily oriented perpendicular to the plane of the substrate. We postulated that the surface density of the compression probe would impact the FRET response, with greater surface density generating an enhanced response (Figure 3F). We found that decreasing probe density leads to a dampened ΔFRET response under T cells. Unfortunately, we are unable to increase the surface density of the M-CRUSH probe using the biotin–streptavidin anchoring chemistry beyond the values tested here (Figure 3G). Future work using different immobilization chemistry to increase local density should improve the M-CRUSH response to compression forces.

A possible contribution to the weaker ΔFRET signal in cell measurements compared to PEG crowding input is the insertion of the organic dyes into the plasma membrane as has been documented by others and particularly for Atto647N.^33,34^ Total Internal Reflection Fluorescence (TIRF) M-CRUSH measurements showed similar signal intensities (Figure S13), suggesting that other parameters need to be optimized to achieve greater S/N.

Finally, we note that the M-CRUSH probe is modular and can be used to investigate compressive forces generated by virtually any cell type. To demonstrate this point, we used human platelets and added these cells to surfaces presenting the 20 °C probe along with cRGD ligand which binds to integrins. Platelets were then mechanically activated using adenosine diphosphate (ADP).^35^ We observed an increase in compression signal when ADP concentration was increased, meanwhile there was little to no change in cell spreading area (Figure S14).

In summary, the M-CRUSH probe is shown to be sensitive to both molecular crowding and interfacial compression. The sensitivity of this probe may be further optimized for different force thresholds by tuning the folding stability of the hairpin. This work provides a vital addition to the tools available for studying mechanical forces exerted by cells. Applications of M-CRUSH may expand our understanding of T cell mechanotransduction as well as a wide array of biological processes.

## Supporting Information Available

Additional experimental details, materials, and methods; full DNA sequences; supplementary figures and notes; and additional references. (PDF)
