# Supplementary material for: Molecular Compressive Force Sensor for Mapping Forces at the Cell–Substrate Interface
Source: J Am Chem Soc. 2024 Feb 28;146(10):6830–6. doi: 10.1021/jacs.3c13648 (PMC10941184; doi:10.1021/jacs.3c13648)
Supplement: Supplementary file 1 — ja3c13648_si_001.pdf [file ja3c13648_si_001.pdf]

Molecular Compressive Force Sensor for Mapping Forces at the Cell-Substrate Interface

Sarah Al Abdullatif[†], Steven Narum[‡], Yuesong Hu[†], Jhordan Rogers[†], Rachel Fitzgerald[†], Khalid Salaita[†‡*]

[†]Department of Chemistry, Emory University, 1515 Dickey Drive, Atlanta, Georgia 30322, United States

[‡]Department of Biomedical Engineering, Georgia Institute of Technology and Emory University, Atlanta, Georgia 30322, United States

*Correspondence should be addressed to K.S. (k.salaita@emory.edu)

Table of Contents

Materials and Methods……S3

A. **Reagents**……S3
B. **T cell sourcing**……S3
C. **Instrumentation**……S3
D. **Oligonucleotide preparation**……S3
E. **Assessing thermodynamic properties**……S4
F. **Surface preparation**……S5
G. **Evaluation of T cell activation states**……S6
H. **Imaging surfaces**……S7
I. **Data analysis**……S7

Supplementary Tables……S9

Table S1. Full sequences of DNA strands used…… S9

Supplementary Figures……S13

Figure S1. Characterization of modified oligonucleotides……S13

Figure S2. Example of data analysis workflow……S14

Figure S3. Van't Hoff analysis for 20°C hairpin in 1X PBS……S15

Figure S4. Crowding in solution……S16

Figure S5. Validation of FRET changes through FLIM……S17

Figure S6. M-CRUSH probe surfaces are non-stimulatory to T cells ……S18

Figure S7. Line scan analysis of compression signal under cells……S19

Figure S8. Dynamic images of FRET signal under T cells……S20

Figure S9. Cell spreading area of treated cells……S21

Figure S10. Pre-fixed cells……S21

Figure S11. FRET signal under migratory T cells……S22

Figure S12. Surface density calibration……S23

Figure S13. FRET measurement using TIRF……S23

Figure S14. Measurement of compressive forces exerted by platelets……S24

References……S25

Materials and Methods

A. Reagents. Cy3B-NHS ester was purchased from GE healthcare Bio-Science (Pittsburgh, PA). Atto647N-NHS ester (18373) was purchased from Sigma Aldrich (St. Louis, MO). Milli-Q water was obtained from a Nanopure system with 18.2 M-1 -cm resistivity. All oligonucleotides were purchased from Integrated DNA Technologies (Coralville, IA) and were purified either by reverse phase HPLC or standard desalting. Streptavidin (S000-01) was purchased from Rockland-Inc (Pottstown, PA). ProPlate® Microtiter (204969) are purchased from Thermo-Fisher Scientific. N-hydroxyl succinimide-biotin was purchased from Sigma Aldrich (St. Louis, MO). (3-Aminopropyl) triethoxysilane (APTES, 440140, 99% purity) was purchased from Sigma-Aldrich. Biotinylated pMHC ovalbumin (SIINFEKL) was obtained from the NIH Tetramer Core Facility at Emory University. Cyclo[Arg-Gly-Asp-d-Phe-Lys(Biotin-PEG-PEG)] (PCI-3697-PI) (biotinylated cRGDfk) was acquired from Peptides International (Louisville, KY). Biotinylated mouse ICAM-1 was purchased from Acro Biosystems (Newark, DE). Biotinylated anti-CD3 was purchased from Biolegend (San Diego, CA). CK666 (Cat# ab141231) was purchased from Abcam (Cambridge, United Kingdom). Blebbistatin (Cat# 72402) was purchased from STEMCELL (Vancouver, Canada). Jasplakinolide was purchased from Thermofisher (Waltham, MA). AX-024 was purchased from selleckchem (Houston, TX). Streptavidin (Cat# S000-01) was purchased from Rockland Immunochemicals Inc. (Rockland, NY). All other reagents and materials (unless otherwise stated) were purchased from Sigma-Aldrich and used without purification. All buffers were prepared with 18.2 MΩ nanopure water.

B. T cell sourcing. The OT-1 transgenic mice are housed at the Division of Animal Resources Facility at Emory University. All the experiments were approved and performed under the Institutional Animal Care and Use Committee (IACUC) protocol. Protocol number: PROTO201800239.

C. Instrumentation. The microscope used was NSTORM Nikon microscope (Ti E motorized inverted microscope body), operated by Nikon Elements software, equipped with an Intensilight epifluorescence source (Nikon), a CFI Apo 100X NA 1.49 objective was used. All ultrapure water was obtained from a Barnstead Nanopure water purifying system (Thermo Fisher) that indicated a resistivity of 18.2 MΩ. Nucleic acid purification was performed using a high-performance liquid chromatography (HPLC, Agilent 1100) equipped with a diode array detector. Microvolume absorbance measurements were obtained using a Nanodrop 2000 UV-Vis Spectrophotometer (Thermo Scientific). Mass spectrometry was conducting using a Thermo scientific LTQ Velos Orbitrap MS. Melting curves were obtained using a Horiba Scientific fluorometer with a Newport temperature control unit (model 350B). Fluorescence lifetime microscopy was performed on a Nikon Ti Eclipse Inverted confocal microscope with a Plan Apo Lambda 60×/1.40 Oil objective. The confocal microscope is equipped with a Picoquant Laser Scanning microscope TCSPC Upgrade with SymPhoTime 64. Flow cytometry was conducted using a Beckman Coulter Cytoflex.

D. Oligonucleotide preparation. These methods were adapted from our prior published work[1] but are described here for the benefit of the readership.

All DNA strands were dissolved in water (18.2 MΩ resistivity, used throughout the whole protocol), vortexed and spun down with a tabletop centrifuge. For modified DNA strands (the anchor and donor strands) the volume of water was tuned such that the final concentration reached 1 mM which was validated by using a nanodrop spectrophotometer to measure the absorbance at 260 nm and determine the final concentration based on the extinction coefficient of the oligonucleotide.

The donor strand has an amine modification at the 3’ terminus to conjugate with the donor fluorophore. Cy3B dye was used for this. The following section describes the conjugation between amines and NHS ester dyes. For end users that do not have access to facilities or resources for nucleic acid modification, modified nucleic acids can instead be purchased from custom DNA synthesis vendors that offer bright and photostable dyes.

10x PBS and 1 M $NaHCO_3$ solutions were prepared. The 1 mM amine donor strand solution (10 nmol) was mixed with 10 µL of 10x PBS, 10 µL of 1 M $NaHCO_3$, and 60 µL of H_2O. Finally, 50 µg of Cy3B NHS ester in 10 µL of DMSO immediately before use and added to the mixture for a total reaction volume of 100 µL. The reaction proceeded at room temperature for 1 hour.

The anchor strand has a modification at each terminus, 5' amine and 3' biotin, to conjugate with the fluorophore and to present the biotinylated surface anchor respectively. The Atto647N anchor strand was prepared by conjugating the amine anchor strand with Atto647N NHS ester. The 1 mM amine anchor strand solution (10 nmol) was mixed with 10 µL of 10x PBS, 10 µL of 1 M $NaHCO_3$, and 60 µL of H_2O. Finally, 50 µg of Atto647N NHS ester in 10 µL of DMSO immediately before use and added to the mixture for a total reaction volume of 100 µL. The reaction proceeded at room temperature for 1 hour.

Reaction mixtures were filtered by P2 desalting gel. First, the reaction mixture was diluted with H_2O to a total volume of 300 µL, which is appropriate for the subsequent HPLC purification step. Hydrated P2 gel (650 µL) was added to a centrifugal device and spun down at 18,000 x g for 1 min. We then removed the liquid at the bottom of the device, added the reaction mixture to the column containing P2 gel, spun down at 18,000 x g for 1 min and collected the reaction mixture at the bottom of the device. NOTE: P2 gel should be hydrated with H_2O at least 4 h before use.

The desalted reaction mixture was purified with HPLC using a C18 column designated for oligonucleotide purification, with solvent A: 0.1 M TEAA in H_2O and B: ACN as the mobile phase for a linear gradient elution 10-100% B over 40 min at a flow rate of 0.5 mL/min. The product that was collected has an absorbance peak for the DNA (260 nm) and an absorbance peak for the fluorophore (560 nm for Cy3B or 647 nm for Atto647N) and was then dried in a vacuum centrifugal concentrator overnight. The products were characterized by mass spectrometry. The expected mass was shown to match the calculated mass for each product.

The dried oligo-dye product was reconstituted in 100 µL water. The concentration of the Cy3B ligand strand and Atto647N anchor strand was determined using the nanodrop spectrophotometer. The dye labeling ratio should be approximately 1:1.

The hairpin strand stock solution was made by dissolving the DNA in water to a final concentration of 100 µM. NOTE: The hairpin strand is unmodified and can be directly custom synthesized from a vendor.

E. Assessing thermodynamic properties

1 µM of DNA hairpin, Cy3B donor strand, and Atto 647N anchor strand (1:1:1) were annealed in 1 M NaCl by heating the solution up to 95 °C for 5 min, then gradually cool down by decreasing the temperature to 20 °C over 30 min in a thermal cycler. The DNA constructs were then dissolved in the appropriate buffer solution (1X PBS, or 5%-25% PEG in PBS) reaching a final concentration of 100 nM. This solution was added to a cuvette and inserted into a temperature controlled fluorometer. The sample was then excited at 545 nm while the emission spectrum was measured at each temperature ranging from 10-35°C. As the temperature increased there was a decrease in emission at 673 nm and an increase at 573 nm (corresponding to Atto 647N and Cy3B respectively). The ratio of these peaks was used to calculate the fraction of DNA unfolded at each temperature. When the acceptor emission divided by total emission was at its highest, the hairpin was assumed to be completely folded; while this ratio at its lowest indicated a completely unfolded hairpin. The thermodynamic properties were then assessed using the following set of equations known as the Van't Hoff analysis:

$$\ln(K) = ln\frac{[\%\ closed]}{[\%\ open]} = -\frac{\Delta H^0}{R}\frac{1}{T} + \frac{\Delta S^0}{R} \qquad (S1)$$

$$\Delta G^0 = \Delta H^0 - T\Delta S^0 \qquad (S2)$$

$$T = Tm \text{ when } \Delta G = 0 \qquad (S3)$$

This analysis was used to determine the melting temperature for each sequence our library. For the 20 °C pseudo-stable hairpin, this analysis was repeated with the following concentrations of 8K PEG in 1X PBS: 5%, 10%, 15%, 20%, and 25%. For each concentration of PEG, ΔG_{PEG} was subtracted from ΔG_{PBS} to give ΔΔG. These values were then plotted as a function of PEG concentration. Using the following equation, we were able to calculate the change in excluded volume (ΔV_{ex}), the change in length (ΔL), and the depletion forces generated by crowding.

$$\Delta\Delta G = k_B T \times C_{PEG} \times \Delta V_{ex} = F_{depl} \times \Delta L \qquad (S4)$$

The method for quantifying ΔΔG, excluded volume (ΔV_{ex}), the change in length (ΔL), and the depletion forces (F_{depl}) was adapted from Castro et al[2].

The change in length (ΔL) was compared to the expected length of the single stranded region of the M-CRUSH hairpin, which was calculated using the worm-like chain model. The mean squared end-to-end distance was calculated using equation S5[3], where L is the contour length and ξ is the persistence length.

$$< R^2 > = 2\xi^2\left(\frac{L}{\xi} - 1 + e^{\frac{-L}{\xi}}\right) \qquad (S5)$$

For single stranded DNA at 100 mM NaCl, the persistence length is estimated to be 2.5 nm, and the contour length is 25.01 nm[4]. This means that the end-to-end distance is approximately 10.6 nm.

F. Surface preparation. Surface preparation protocols were adapted from our previously published work[5], but is described below for convenience.

Day 1

Glass coverslips were placed on a polytetrafluoroethylene rack in a 50 mL beaker then submerged in ethanol and sonicated for 10 min in ultrasonics cleaner (operating frequency 35 KHz). After sonication, the liquid was discarded and the beaker with the rack and coverslips was placed in an oven to remove any remaining organic solvent.

Piranha solution was prepared by slowly adding hydrogen peroxide to sulfuric acid in the ratio of 1:3. To etch the glass coverslips, the rack was then transferred to the beaker containing Piranha solution and incubated for 30 minutes. The rack was then moved back to a clean beaker and rinsed with water at least 6 times.

CAUTION: Organic substances react vigorously with Piranha solution and may cause an explosion. Be careful and always work with Piranha solution in a fume hood. Make sure to wear a labcoat, thick gloves, and safety goggles. Never store fresh Piranha solution in a sealed container.

NOTE: The hydrogen peroxide to sulfuric acid ratio should be kept under 1:2 (v:v) and should never exceed 1:1. When submerging the rack with coverslips in Piranha solution, place them in the solution slowly and carefully. Do not discard the solution immediately after etching, as it is still active and hot. Leave it in the beaker overnight before pouring it in the acid waste container.

The rack was then rinsed 3 times with ethanol to remove the water, then immersed in 3% aminopropyl triethoxy silane (APTES) (v/v) in 40 mL of ethanol and incubated for 1 hour.

After rinsing the surfaces 6 times by submerging them into ethanol, they were dried in an oven at 80 °C for 20 min. To modify the amine groups on the coverslips, the glass slides were coated with 2.5 mg/mL of Biotin NHS ester in DMSO.

Day 2

Glass cover slips were washed with ethanol and dried before mounting onto ProPlate 96 well plate with adhesive backing. Each well was rinsed with water, then blocked with 1% BSA for 30 minutes. Then, 40 µg/mL of streptavidin in 1x PBS was incubated on the coverslips for 30 min at room temperature. The wells were rinsed with PBS 3 times after incubation to wash away the excess amount of streptavidin.

Finally, pre-hybridized DNA probes were incubated on the surfaces for 1 hour at room temperature at a concentration of 100 nM. For live cell imaging, biotinylated anti-CD3 was

mixed into the probe solution before adding the DNA to surfaces (final concentration = 10 μg/mL in each well).

G. Evaluation of T cell activation states

160,000 Cells were incubated on glass slides in 200 μL of media (inactivated) and in some cases with 10 μg/mL of anti-CD3 and anti-CD28 (activated) or M-CRUSH probe immobilized on surfaces. Activated and inactivated cells were incubated at 37 °C for 4 hours. The experimental group was incubated on DNA modified surfaces for 1 hour at room temperature, then transferred to 37 °C for 3 additional hours. Then, all cell populations were stained by incubating with PE-Cy5 modified anti-CD69 on ice for 30 minutes. Cells were then fixed and analyzed by flow cytometry to evaluate the CD69 expression levels which correspond to the activation state.

H. Imaging surfaces

Surfaces were imaged in three fluorescence channels: donor (ex: 545 nm, em: 605 nm), acceptor (ex: 620 nm, em: 700 nm), and sensitized FRET (ex: 545 nm, em: 700 nm) using epifluorescence microscopy.

For crowding experiments:

Each DNA sequence was imaged in 1x PBS, 10%PEG, and 25% PEG buffer solutions.

For cell experiments:

OT-1 CD8+ naïve T cells were purified from the spleens of sacrificed mice using the MACS mouse CD8+ T cell isolation kit with a MACS separator following manufacturer's instructions, then resuspended in HBSS at 2 x 10^6 cells/mL and kept on ice prior to use[1].

For drug screening:

After purification and before plating, cells were treated with each of the following for 10-15 minutes, then added to surfaces: AX-024 (500 nM), blebbistatin (50 μM), ck666 (50 μM), jasplakinolide (500 nM), and latrunculin B (5 μM).

Cells fixed in solution were incubated with 3% formaldehyde for 10 minutes, then centrifuged and resuspended in 1X PBS.

Purified cells were then plated onto surfaces at ~100 x 10^5 - 200 x 10^5 density and allowed to attach and spread for ~25 min at room temperature. Fluorescence signals in the Cy3B, Atto 647N, and sensitized FRET channels were imaged using a 100x objective.

NOTE: Be sure to use consistent gain and exposure time across all 3 fluorescence channels.

I. Data analysis

NOTE: Image analysis was performed using Fiji software, and the quantitative analysis was performed using analysis software.

To begin, detector noise was subtracted from all fluorescence channels. Detector noise was determined by imaging a sample containing only buffer, without the presence of any fluorescent dye. The fluorescence signal detected in each channel is determined to be the detector noise signal.

Then donor and acceptor bleedthrough was removed from the sensitized FRET channel. To quantify bleedthrough, a sample containing only the donor, and a sample containing only the acceptor were imaged in all three channels. Donor bleedthrough is determined by the signal ratio in the sensitized FRET channel to the donor channel, and acceptor bleedthrough is the ratio of signal in the sensitized FRET channel to acceptor channel. These ratios give a constant value shown in equation S6, where α is the donor bleedthrough constant, I_{fd} is the fluorescence intensity of the donor only sample in the sensitized FRET channel, I_{dd} is the fluorescence intensity of the donor only sample in the donor channel, β is the acceptor bleedthrough constant, I_{fa} is the fluorescence intensity of the acceptor only sample in the sensitized FRET channel, and I_{aa} is the fluorescence intensity of the acceptor only sample in the acceptor channel.

$$\alpha = \frac{I_{fd}}{I_{dd}} \qquad \beta = \frac{I_{fa}}{I_{aa}} \qquad \text{(S6)}$$

The sensitized FRET signal from the experimental sample was then adjusted to give the corrected FRET (F_c) by subtracting the product of the bleedthrough constant and the intensity in the donor and acceptor channels, respectively. This is shown in equation S7, where I_{fe} is the fluorescence intensity of the experimental sample in the sensitized FRET channel, I_{de} is the fluorescence intensity of the experimental sample in the donor channel, and I_{ae} is the fluorescence intensity of the experimental sample in the acceptor channel.

$$F_c = I_{fe} - \alpha I_{de} - \beta I_{ae} \qquad \text{(S7)}$$

Afterwards, the signal was converted to a FRET index by taking the ratio of the corrected FRET to the sum of the donor and corrected FRET signal, shown in equation S8. This was done for each image using the image expression parser function on Fiji.

$$FRET\ Index = \frac{F_c}{I_{de}+F_c} \times 100 \qquad \text{(S8)}$$

With the exception of figure S2, all representative images are shown after subtracting detector noise and donor and acceptor bleedthrough.

The FRET index was averaged from multiple background regions. We drew ROIs of cells on RICM (reflection interference contrast microscopy) images with Image J Freehand Selections tool. The FRET index under cells was also averaged. We then subtracted the background FRET from this signal to give ΔFRET. The measurements were then exported for quantitative analysis with analysis software.

Supplementary Tables

Table S1. Full sequences of DNA strands used

**Strand name**	**Sequence (5’-3’)**	**Structure of modifications**
Anchor strand	/C6 Amine/CGCATCTGTGCGGTATTTCACTTT/Biotin/	C6 Amine: 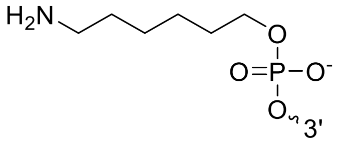 Biotin: 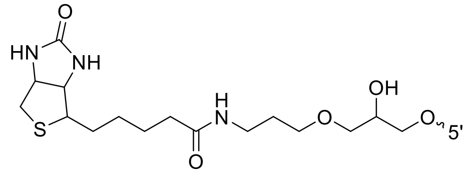
Donor strand	TTTGCTGGGCTACGTGGCGCTCTT/Amine/	Amine: 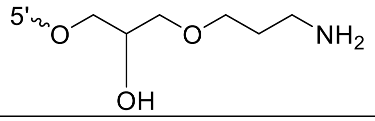
**Strand name**	**Sequence (5’-3’)**	**Minimum free energy structure at room temperature** 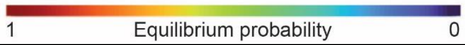
Poly T	GTGAAATACCGCACAGATGCG TTT TTT TTT TTT TTT TTT TTT TTT TTT TTT T AAGAGCGCCACGTAGCCCAGC	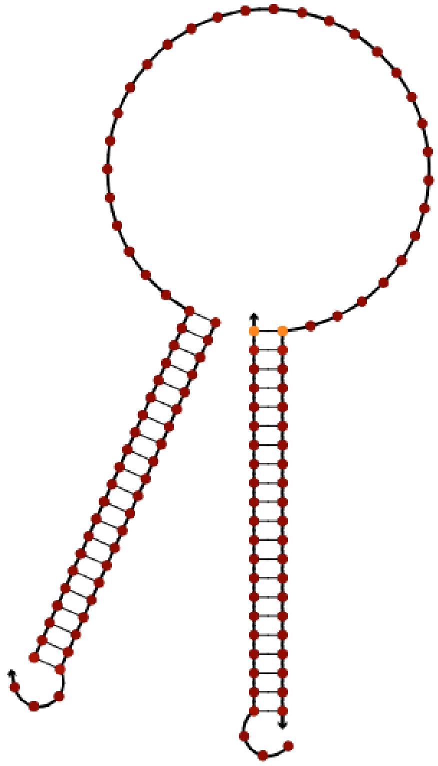

13 °C	GTGAAATACCGCACAGATGCG TTT ATA TTT TTT TTT TTT TTT TTT TTT TTT ATA TTT T AAGAGCGCCACGTAGCCCAGC	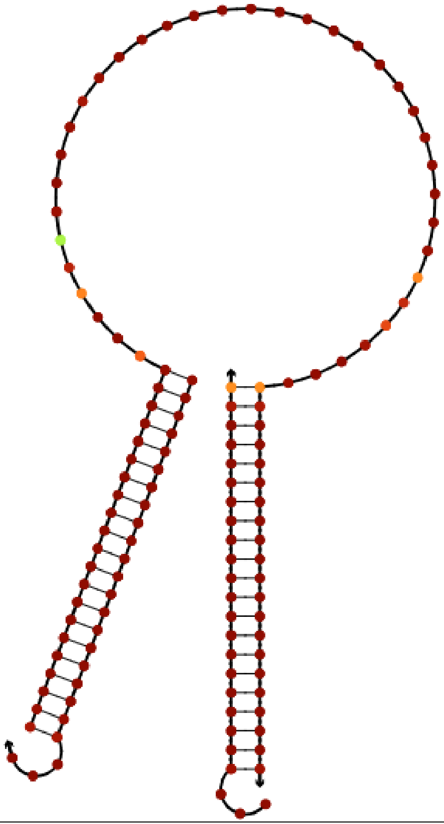
20 °C	GTGAAATACCGCACAGATGCG TGT TTA TTT GTT TTT TTT TTT TTT TTC ATA TAT ACT AAGAGCGCCACGTAGCCCAGC	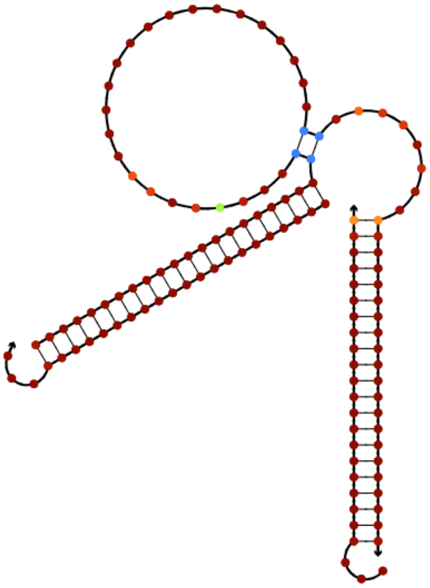

29 °C	GTGAAATACCGCACAGATGCG TTT CTA TAT ATA TTT TTT TTA TTT ATA GTT T AAGAGCGCCACGTAGCCCAGC	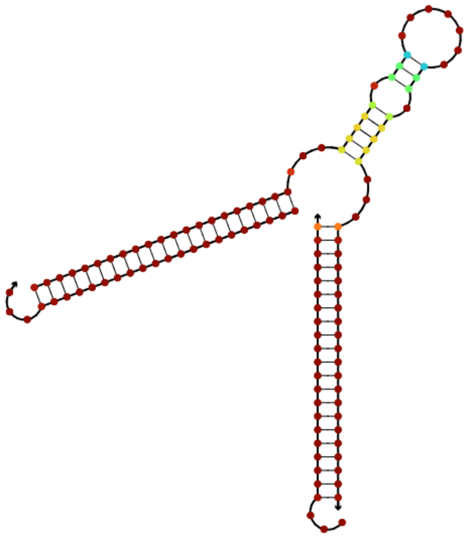
33 °C	GTGAAATACCGCACAGATGCG TTTG TAT ATA TGT TTT TTT CAT TTA TAC TTT AAGAGCGCCACGTAGCCCAGC	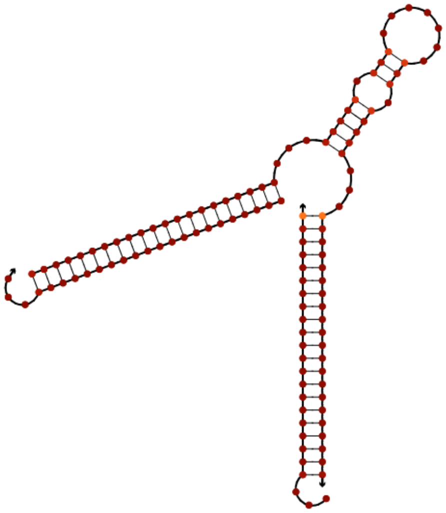

38 °C	GTGAAATACCGCACAGATGCG TTA TAT AAA TAT TTT TTT TAT TTA TAT TTT AA AAGAGCGCCACGTAGCCCAGC	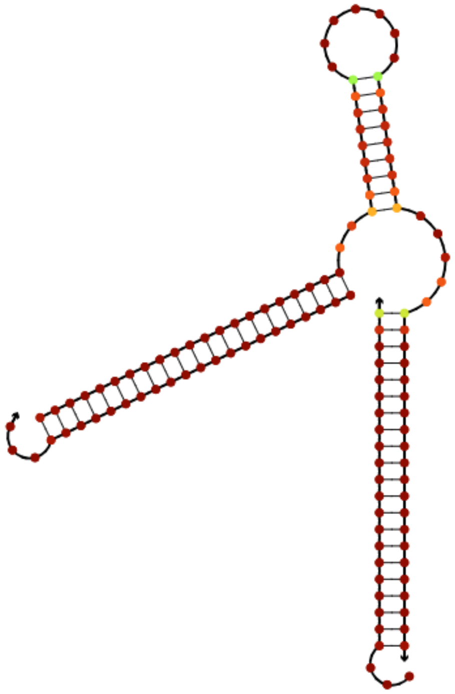
60 °C	GTGAAATACCGCACAGATGCGTTTGTATA AATGTTTTTTTCATTTATACTTTAAGAGCG CCACGTAGCCCAGC	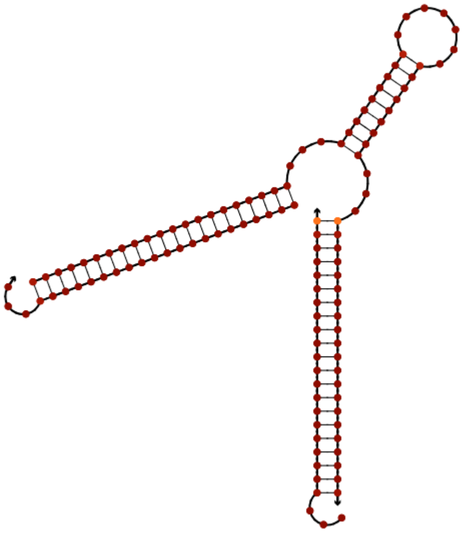

Supplementary Figures

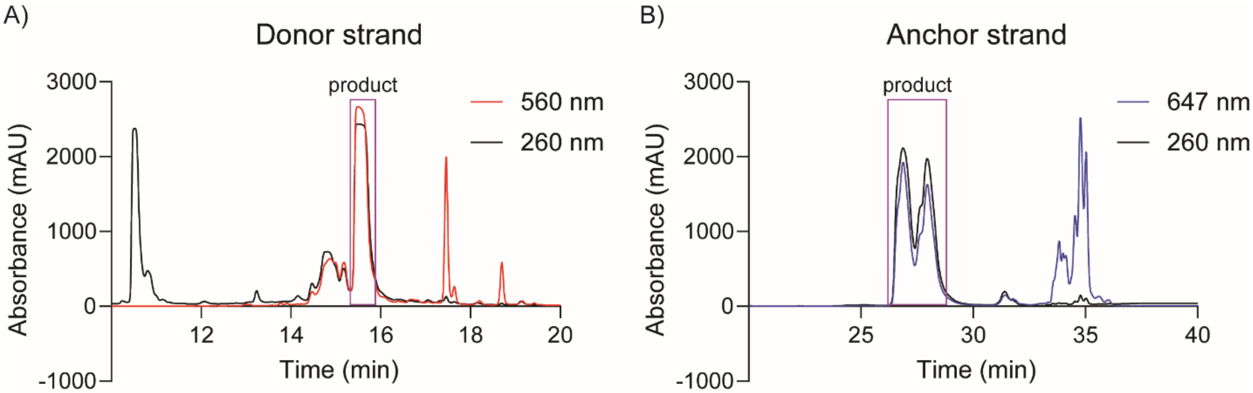

C)

	Expected mass	Calculated mass
**Donor strand**	8545.4 g/mol	8544.7 g/mol
**Anchor strand**	8293 g/mol	8290.9 g/mol

Figure S1. Characterization of modified oligonucleotides. A) and B) HPLC traces of donor and anchor strands, respectively. The donor strand is modified with Cy3B, and the anchor strand is conjugated to Atto 647N. Boxes indicate the reaction product associated with each chromatogram. C) Molecular weight of each isolated product as calculated by mass spectroscopy.

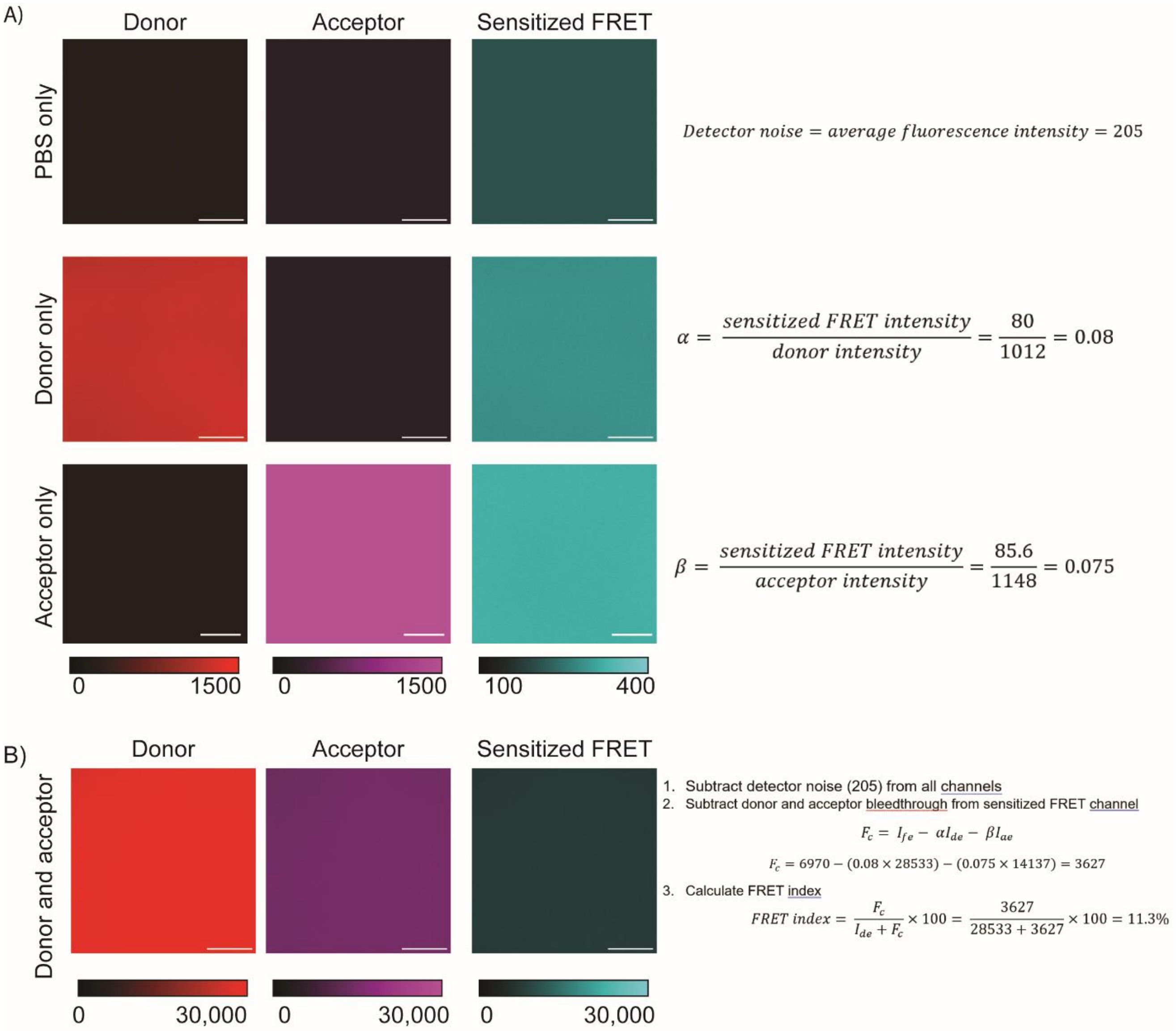

Figure S2. Example of data analysis workflow. Further description of how this analysis was conducted is detailed in the Data Analysis portion of the Materials and Methods section. A) Quantification of detector background noise (top) as well as donor (middle) and acceptor (bottom) bleedthrough constants. Scale bar = 5 μm. B) Calculation of FRET index including detector noise and bleedthrough subtraction. Scale bar = 5 μm.

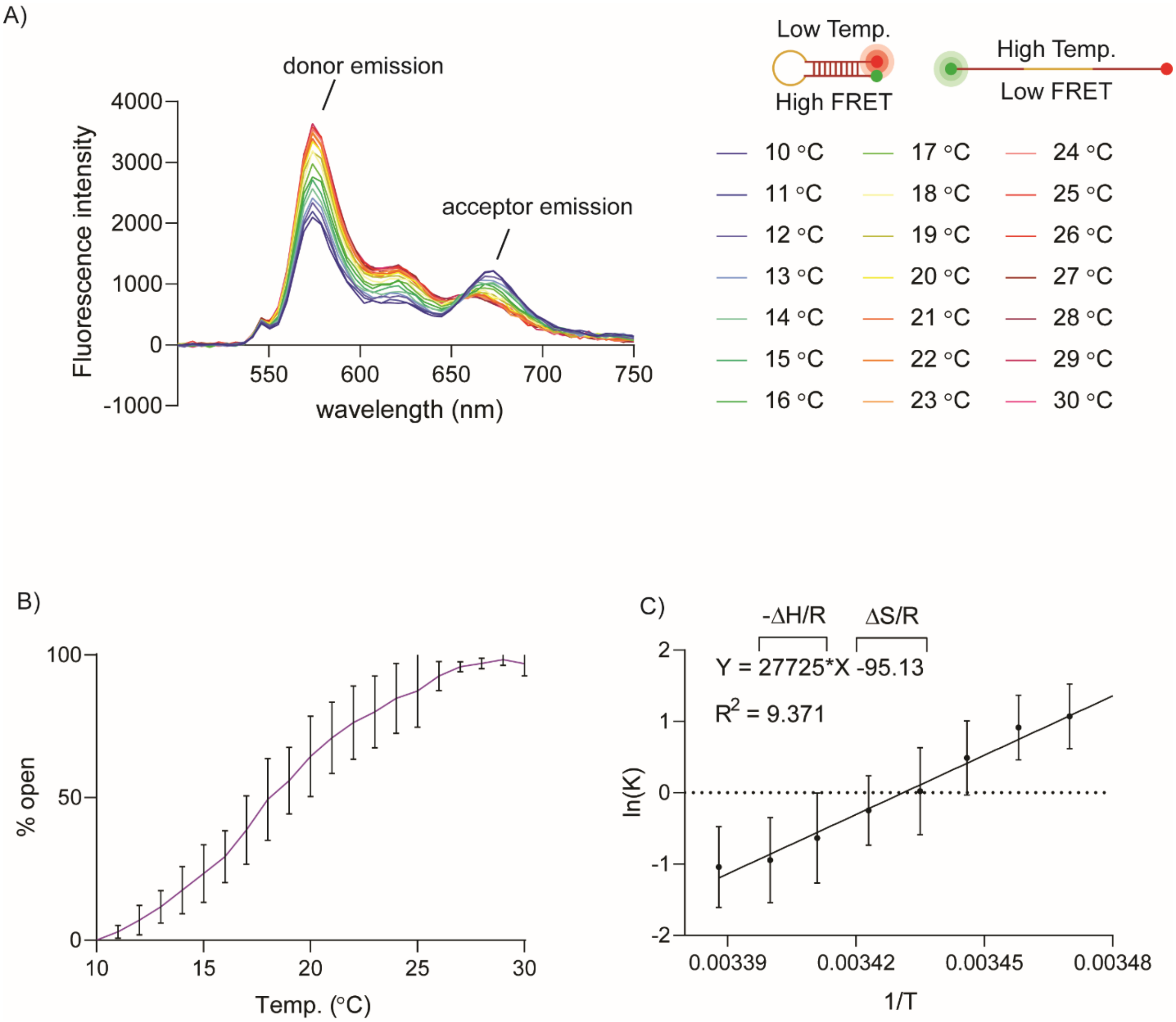

Figure S3. Van't Hoff analysis for 20°C hairpin in 1X PBS. A) Fluorometer emission spectrum for 20°C hairpin in 1X PBS over temperatures ranging from 10-30 °C. Excitation peak is at 545 nm. Donor emission peak is at 573 nm. Acceptor emission peak is at 670 nm. B) Melting curve for 20°C hairpin in 1X PBS. Fraction open is proportional to donor emission/acceptor emission. C) Van't Hoff analysis based on melting curve. Linear regression gives the enthalpy and entropy through the slope and x-intercept respectively. Free energy and melting temperature are calculated based on these values. Error bars represent the standard error of the mean from triplicate thermal melt measurements.

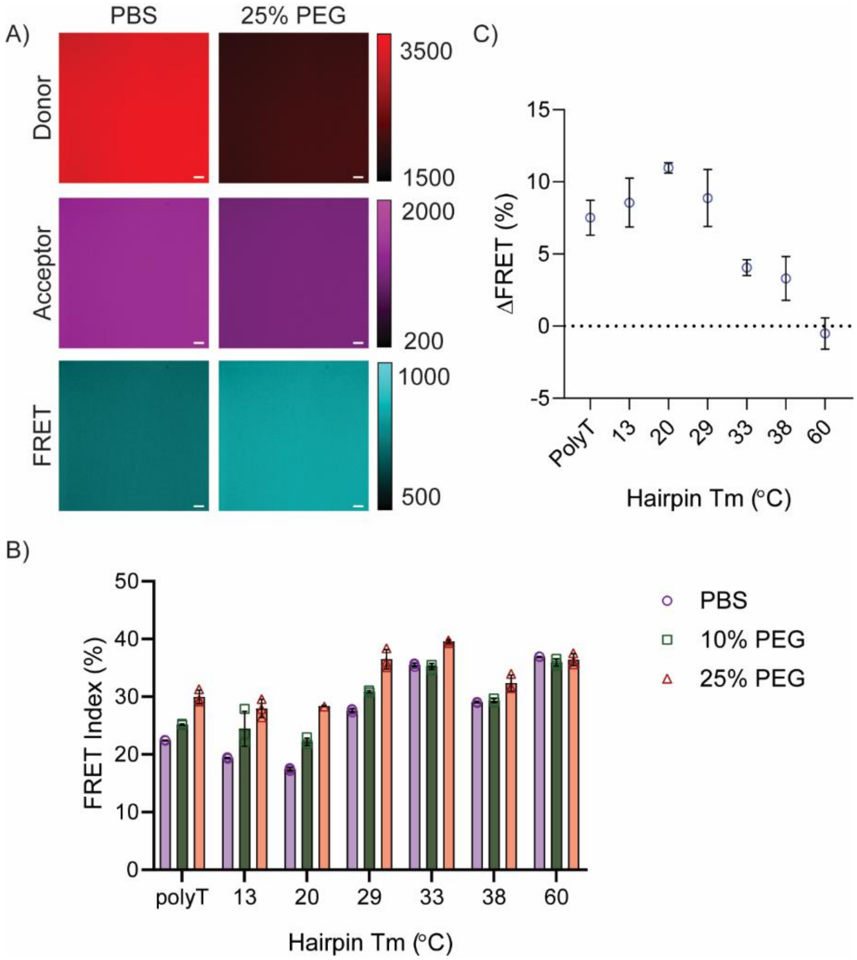

Figure S4. Crowding experiment in solution. A) 20 °C hairpin in PBS (left) and 25% PEG in PBS (right) 3 channel fluorescence imaging. Addition of crowding agent resulted in decrease of donor fluorescence and increase of sensitized FRET. Scale bar = 5 µm. B) FRET indices for full range of hairpins in PBS, low crowding environment (10% PEG) and high crowding environment (25% PEG). C) Change in FRET between 25% PEG and PBS. Error bars represent the standard error of the mean from triplicate measurements.

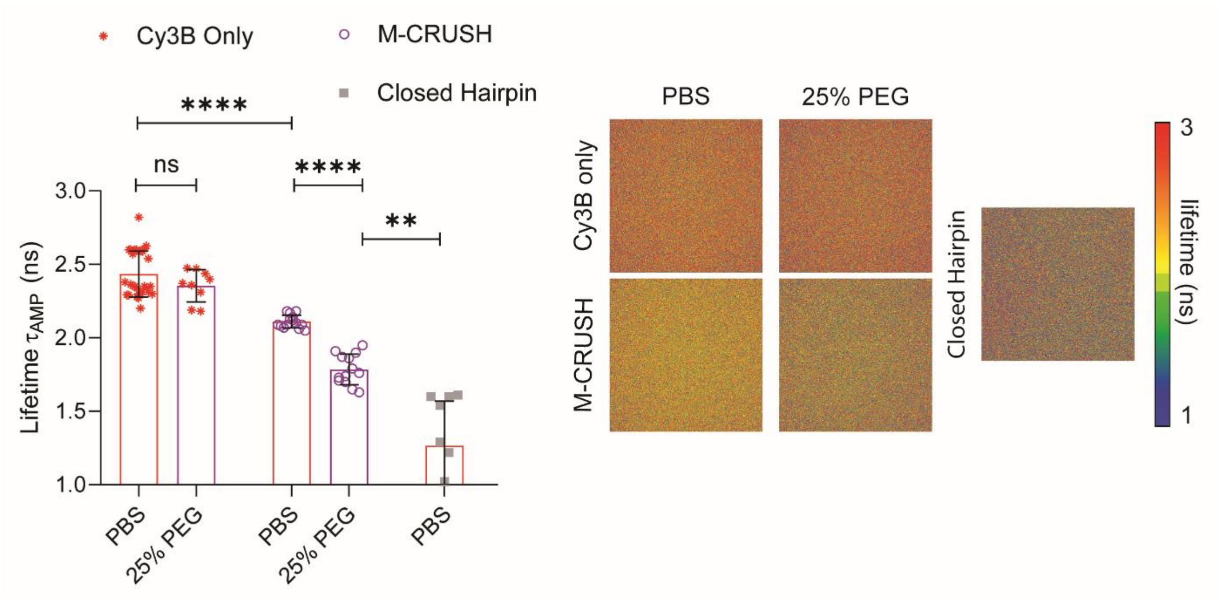

Figure S5. Validation of FRET changes through FLIM. The lifetime of the donor (Cy3B) was measured on different DNA hairpins. Donor only control displayed lifetime ~2.5 ns in PBS and 25% PEG. M-CRUSH probe showed lower lifetime due to presence of acceptor. Lifetime is further lowered in crowded environment due to hairpin folding. Cy3B on the fully closed hairpin (60 °C hairpin) was most quenched by the acceptor and thus exhibited minimum Cy3B lifetime. Protocols for measuring and fitting fluorescence lifetimes can be found in our group's previously published work[6]. Error bars represent the standard error of the mean from triplicate surface measurements. A one-way ANOVA was conducted to determine statistical significance. P values are indicated as follows: 0.5 (ns), 0.05 (*), 0.01(**), 0.001 (***), <0.0001 (****).

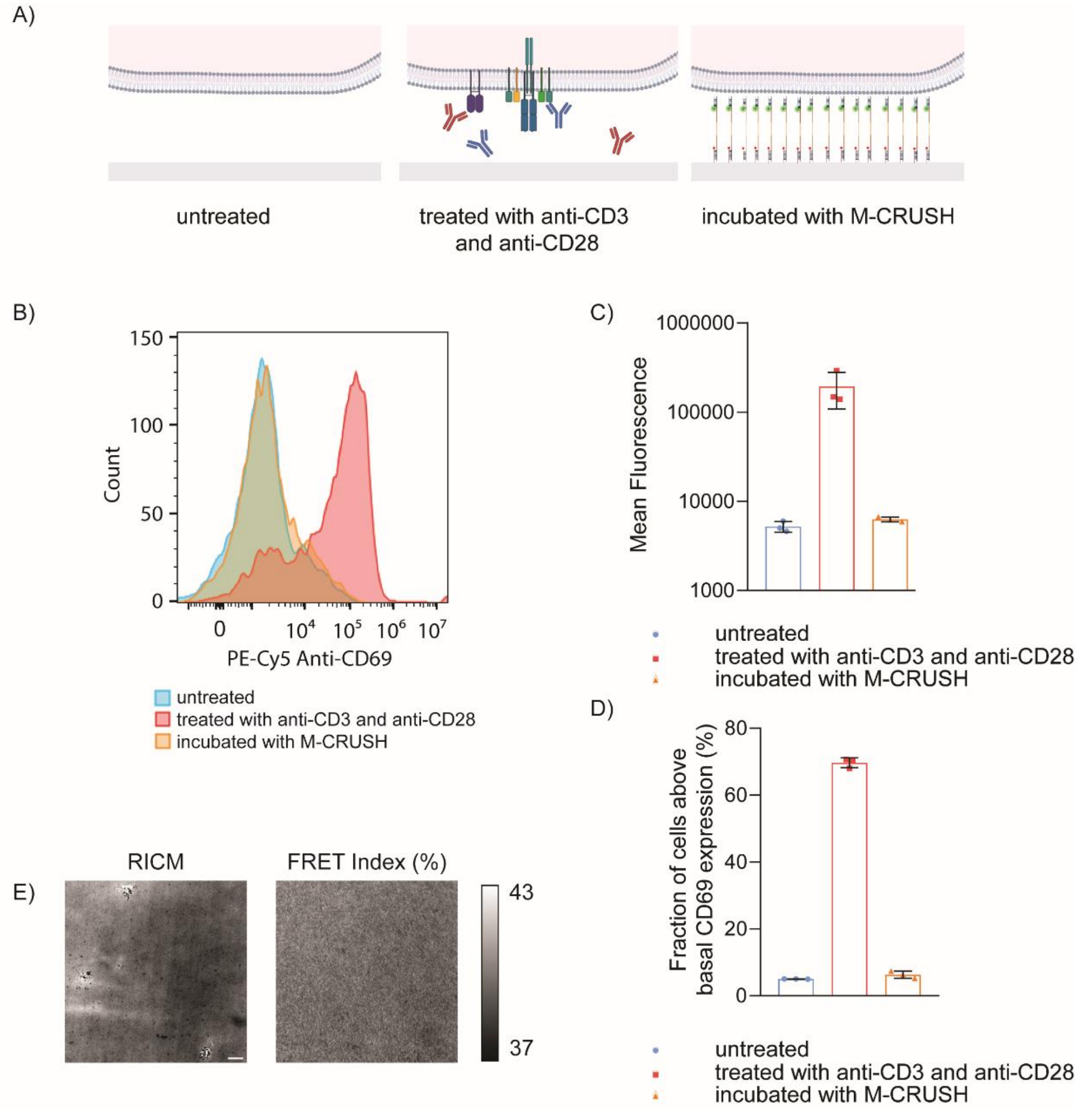

Figure S6. M-CRUSH probe surfaces are non-stimulatory to T cells. A) Schematic representing experimental setup. The negative control group were T cells plated onto unmodified glass slides, while the positive control group included identical T cells that were treated with stimulatory antibodies (anti-CD3 and anti-CD28). These two experimental groups were compared against T cells seeded onto surfaces modified with the M-CRUSH probe. B) Representative data from flow cytometry measurements. Untreated cells and cells incubated with M-CRUSH show basal CD-69 expression levels, while cells treated with antibody show an increase of CD69 up to 3 orders of magnitude. C) Average fluorescence intensity for each condition. Each data point represents the mean of an entire cell population from a single animal. D) Fraction of cells exceeding basal CD69 expression, which was identified via thresholding the untreated data at the 95th percentile. Each data point represents the result from a single animal. In C) and D), Error bars represent the SD from n=3 biological replicates. E) Cells incubated on surfaces modified with M-CRUSH only, lacking the presence of a ligand. RICM (left) and FRET signal (right) displays lack of interaction between the surface and cells. Scale bar = 5 µm.

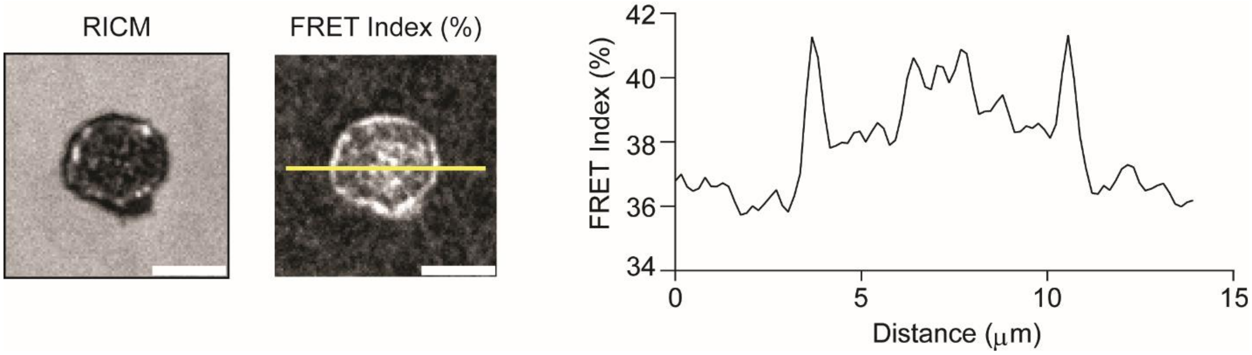

Figure S7. Line scan analysis of compression signal under cells. Up to 6% increase in FRET index was observed at the perimeter of the cell-surface contact zone as well as in a subset of puncta under the cell.

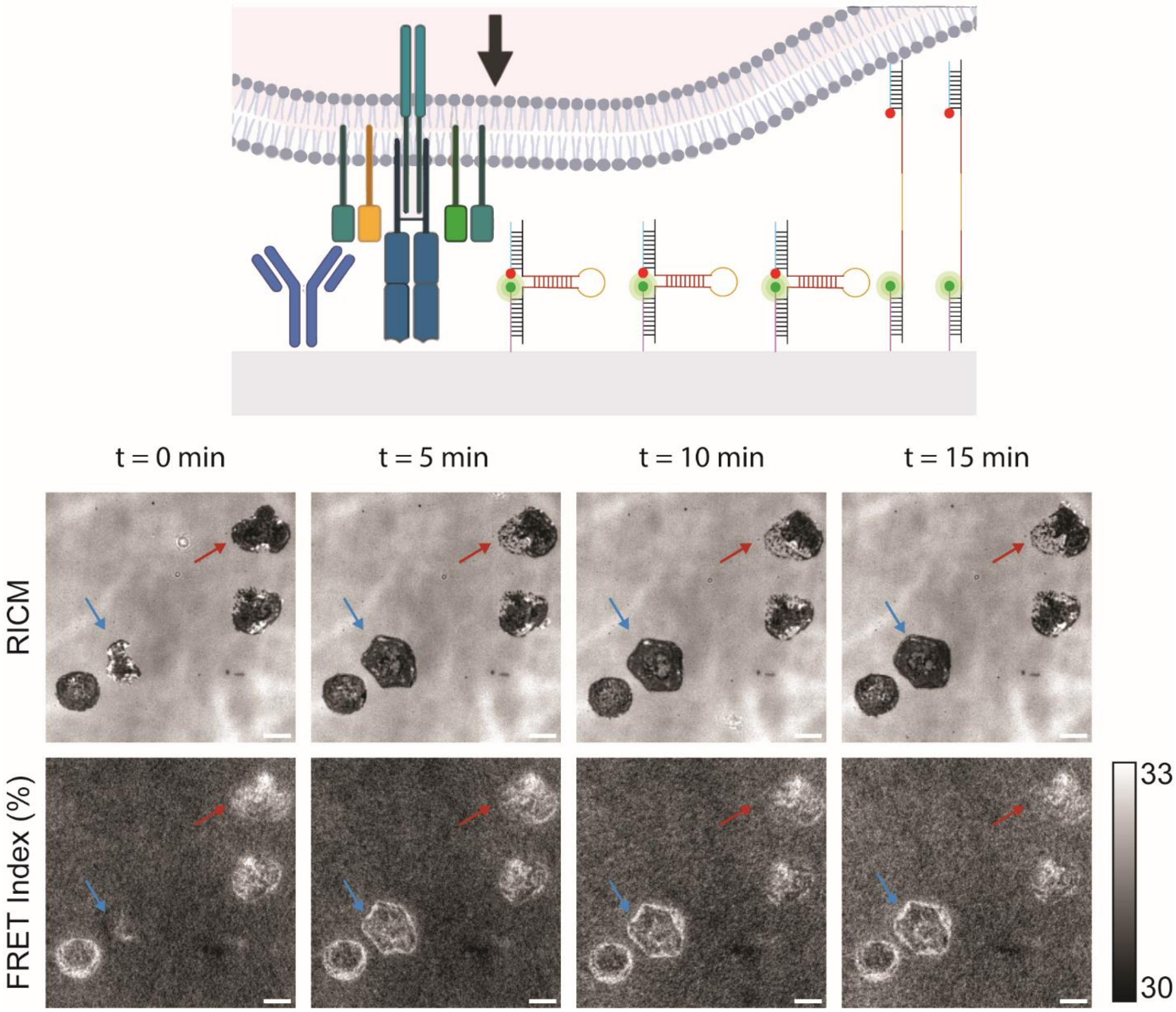

Figure S8. Dynamic images of FRET signal under T cells. Signal appeared as the cell landed and spread on the surface (blue arrow). The signal shape also changed along with the cell morphology (red arrow). Scale bar = 5 μm.

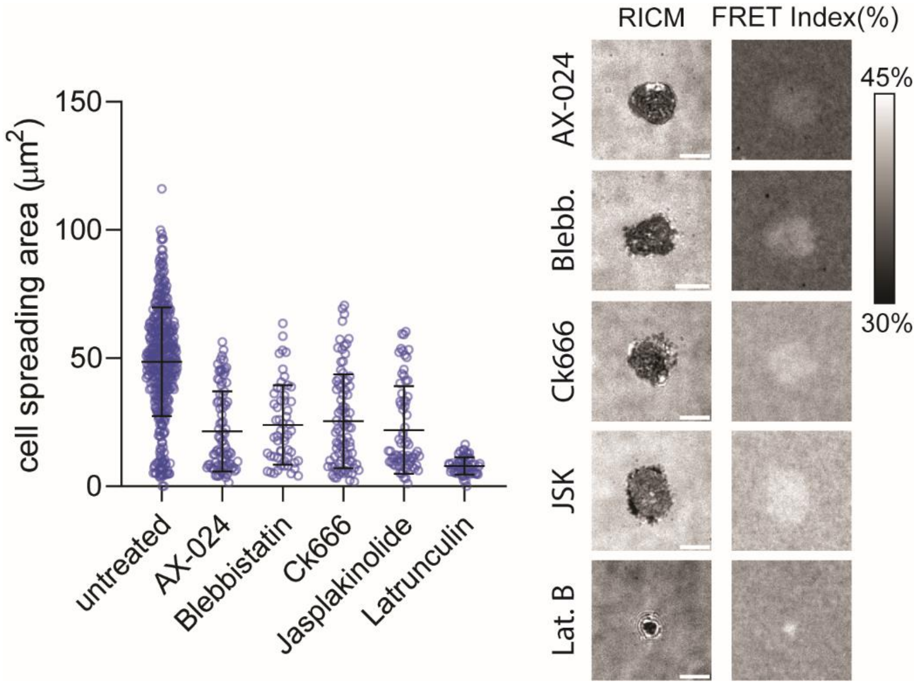

Figure S9. Cell spreading area of treated cells. Acto-myosin inhibitors result in a reduction of cell spreading area which may play a role in decreased FRET signal. Scale bar = 5 µm. Each data point represents a single cell. Error bars show the standard error of the mean from n=3 biological replicates.

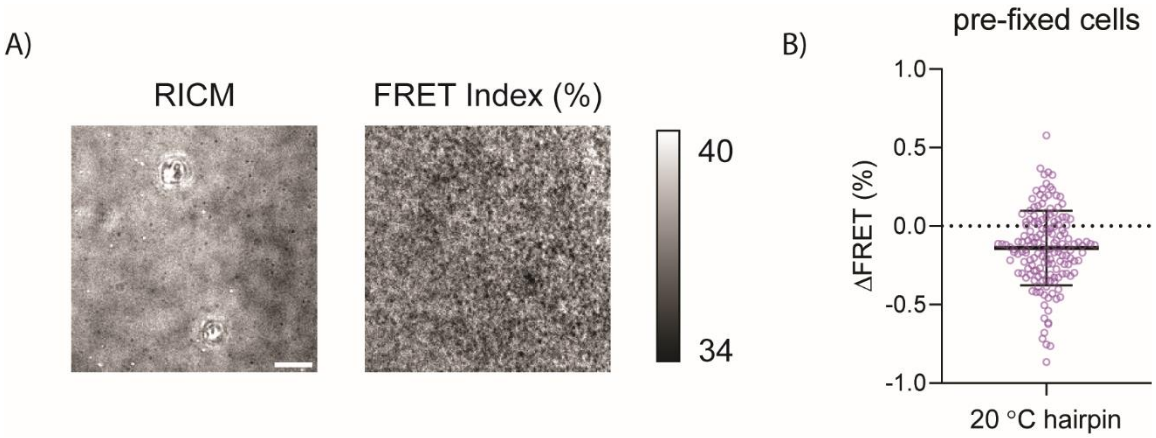

Figure S10. Pre-fixed cells. T cells have been fixed in solution prior to plating on surfaces presenting M-CRUSH and anti-CD3. A) Representative images showing that the cell binds to the surface but does not spread. B) Ensemble data shows that fixed cells do not generate FRET signal. Scale bar = 5 µm. Each data point represents a single cell. Error bars show the standard error of the mean from n=3 biological replicates.

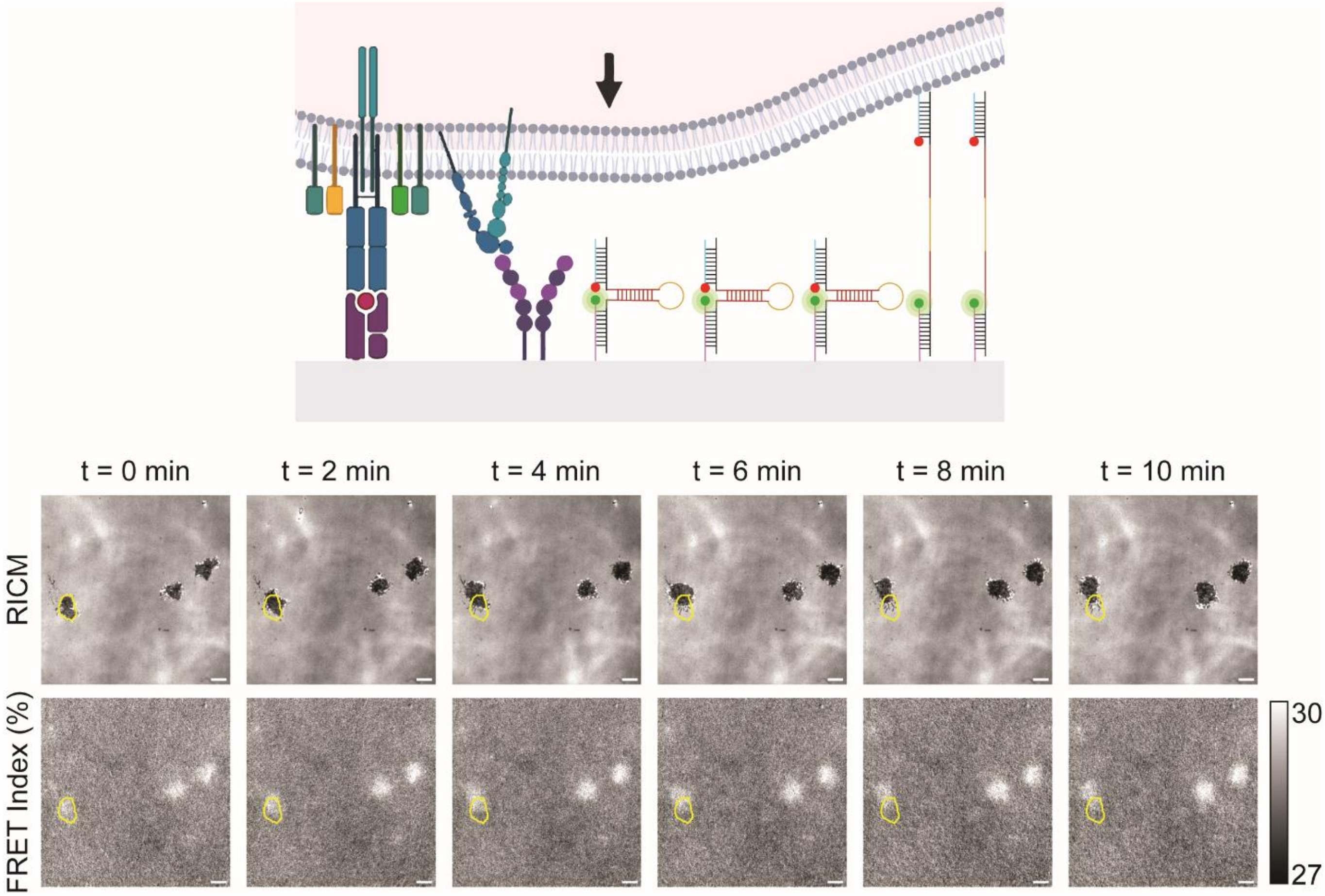

Figure S11. FRET signal under migratory T cells. Migratory T cells moved across surfaces presenting pMHC and ICAM. Signal in FRET index also moved accordingly. The starting position of the cell is outlined in yellow. Scale bar = 5 μm.

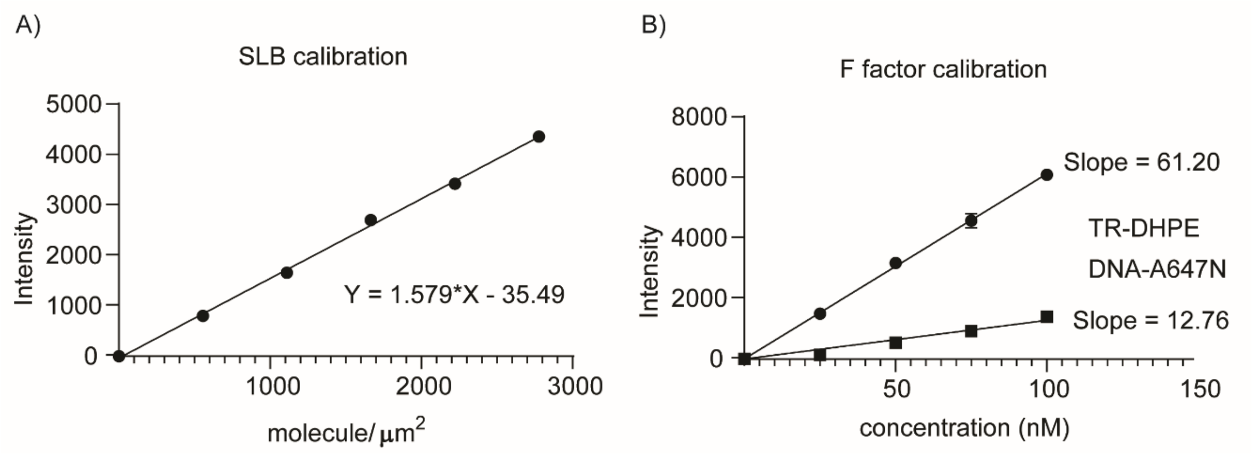

Figure S12. Surface density calibration. A) Plot of fluorescence intensity versus Texas Red labeled phospholipid (TR-DHPE) per micron within the SLB. This plot was used to convert the fluorescence intensity measurements into molecular density. B) Plot of fluorescence intensity versus concentration for TR-DHPE vesicles and Atto647N-DNA. These measurements were performed in solution. F factor was calculated as the ratio of Atto647N to Texas Red fluorescence. The F factor was used in combination with the SLB calibration to determine the density of probes on the surface[1].

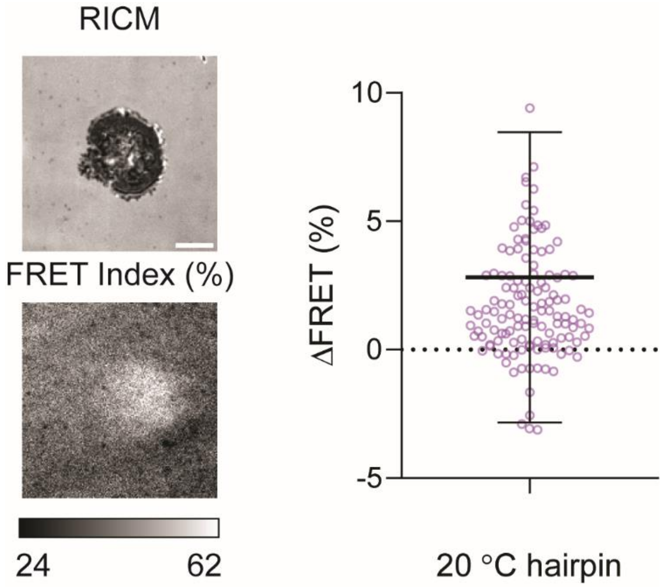

Figure S13. FRET measurement using TIRF. Sample was excited with a 562 nm laser. Emission was collected in two channels and filtered at 561 nm (donor) and 700 nm (FRET) respectively. Scale bar = 5 μm. Each data point represents a single cell. Error bars show the standard error of the mean from n=3 biological replicates.

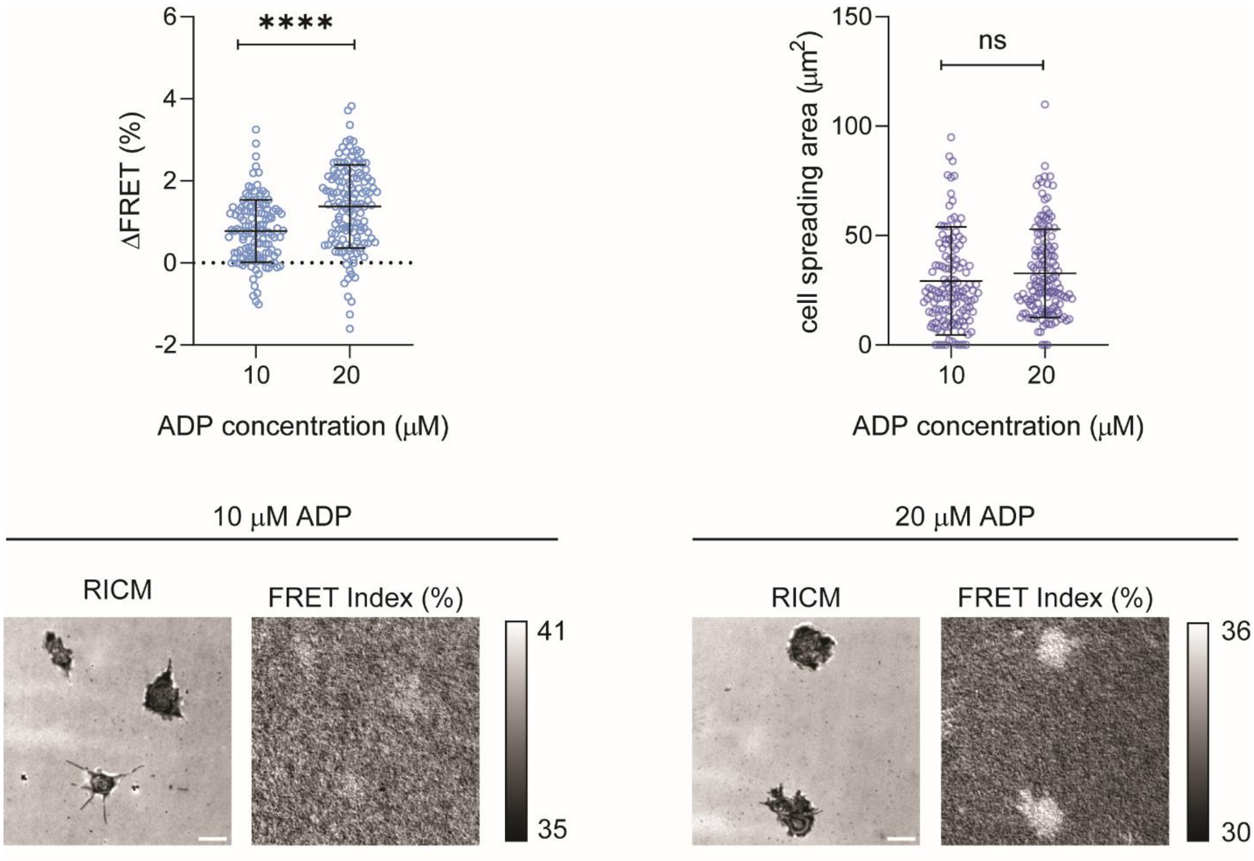

Figure S14. Measurement of compressive forces exerted by platelets. Surfaces were prepared by incubating a binary mixture of the 20 °C M-CRUSH probe and the biotinylated cRGDfk peptide for 1 hour at a 10:1 concentration ratio with the final DNA concentration being 100 nM. Human platelets were collected and isolated as reported in our previous work[7]. Platelets were then added to the rinsed surfaces at a density of ~ 5 x 10^6 cells/mL, and mechanically activated using ADP. Fluorescence measurements were made after allowing the cells to spread on the surfaces for 15 minutes. Higher concentrations of ADP resulted in a greater increase of FRET signal without significant changes to spreading area. Scale bar = 5 µm. Each data point represents a single cell. Error bars show the standard error of the mean from n=3 biological replicates. P values are indicated as follows: 0.5 (ns), <0.0001 (****).

References

(1) Hu, Y.; Ma, V. P. Y.; Ma, R.; Chen, W.; Duan, Y.; Glazier, R.; Petrich, B. G.; Li, R.; Salaita, K. DNA-Based Microparticle Tension Sensors (MTS) for Measuring Cell Mechanics in Non-Planar Geometries and for High-Throughput Quantification. *Angewandte Chemie International Edition* **2021**, *60* (33), 18044–18050.

(2) Hudoba, M. W.; Luo, Y.; Zacharias, A.; Poirier, M. G.; Castro, C. E. Dynamic DNA Origami Device for Measuring Compressive Depletion Forces. *ACS Nano* **2017**, *11* (7), 6566–6573.

(3) Milstein, J.; Meiners, J.-C. *Encyclopedia of Biophysics*; 2013.

(4) Murphy, M. C.; Rasnik, I.; Cheng, W.; Lohman, T. M.; Ha, T. Probing Single-Stranded DNA Conformational Flexibility Using Fluorescence Spectroscopy. *Biophys J* **2004**, *86* (4), 2530–2537.

(5) Rao, T. C.; Beggs, R. R.; Ankenbauer, K. E.; Hwang, J.; Pui-Yan Ma, V.; Salaita, K.; Bellis, S. L.; Mattheyses, A. L. ST6Gal-I-Mediated Sialylation of the Epidermal Growth Factor Receptor Modulates Cell Mechanics and Enhances Invasion. *Journal of Biological Chemistry* **2022**, *298* (4), 101726..

(6) Glazier, R.; Brockman, J. M.; Bartle, E.; Mattheyses, A. L.; Destaing, O.; Salaita, K. DNA Mechanotechnology Reveals That Integrin Receptors Apply PN Forces in Podosomes on Fluid Substrates. *Nat Commun* **2019**, *10* (1), 1–13.

(7) Duan, Y.; Szlam, F.; Hu, Y.; Chen, W.; Li, R.; Ke, Y.; Sniecinski, R.; Salaita, K. Detection of Cellular Traction Forces via the Force-Triggered Cas12a-Mediated Catalytic Cleavage of a Fluorogenic Reporter Strand. *Nature Biomedical Engineering 2023 7:11* **2023**, *7* (11), 1404–1418.
